# Long-term outcomes of drainless anatomical lung resection surgery for pulmonary malignancies

**DOI:** 10.1186/s13019-024-03303-8

**Published:** 2025-02-03

**Authors:** Ting-Fang Kuo, Mong-Wei Lin, Ke-Cheng Chen, Shuenn-Wen Kuo, Pei-Ming Huang, Jang-Ming Lee

**Affiliations:** Department of Surgery, National Taiwan University Hospital, National Taiwan University College of Medicine, No. 7, Zhongshan S. Rd., Zhongzheng Dist, Taipei, 100225 Taiwan

**Keywords:** Anatomical lung resection, Drainless, Minimally invasive surgery, Pulmonary malignancy

## Abstract

**Objective:**

Drainless minimally invasive anatomical lung resection surgery for pulmonary malignancies is safe and feasible in terms of early postoperative outcomes. However, the quality of surgery in the long term remains uncertain. This study aimed to investigate the perioperative outcomes, 3-year overall, and disease-free survival rates of patients who underwent minimally invasive anatomical lung resection surgery with the drainless technique for pulmonary malignancies.

**Methods:**

Fifty-eight patients who underwent drainless minimally invasive anatomical lung resection surgery for pulmonary malignancies (36 -lobectomy; 22 -segmentectomy) between November 2017 and June 2022 by a single surgeon were enrolled. Patients’ characteristics and perioperative, early postoperative, and long-term data were collected. The lymph node dissection stations and number, resection margin, 3-year overall and disease-free survival rates were assessed.

**Results:**

The median age was 64 years. Forty-four patients were females (76%) and forty-seven patients were non-smokers (81%). The median five-factor modified frailty index was 1. Most patients had primary lung cancer; four (7%), 43 (74%), seven (12%), and three (5%) had stage 0, I, II, and III, respectively. The median lymph node dissection stations was four, and the number was 17. The resection margin was free in 98% of the cases. The 3-year overall survival rate was 98.3% in all patients, and 97.2% and 100% in the lobectomy and segmentectomy subgroups, respectively. The 3-year disease-free survival rate was 85.3% in all patients and 80.5% and 92.9% in the lobectomy and segmentectomy subgroups, respectively.

**Conclusion:**

The drainless technique is safe and feasible for minimally invasive anatomical lung resection surgery for pulmonary malignancies in terms of early postoperative and long-term outcomes. However, further randomized controlled studies are warranted.

## Introduction

With the evolution of thoracic enhanced recovery after surgery (ERAS^®^), a single small-diameter chest drain with early removal has become possible [1]. Recently, the drainless technique, in which a chest drain is omitted after minimally invasive thoracic surgery, has been reported to be safe and feasible for wedge resection in terms of early postoperative outcomes in selected patients [2–11]. As for anatomical lung resection, the procedure is more complicated, requiring extensive vascular, bronchial, and lymphatic dissection, which results in more residual air space, leading to a higher probability of air leaks or pleural effusion accumulation. This may prevent surgeons from omitting the chest tube for anatomical lung resection. Very few reports are available in the literature on this surgical technique [12–14]. We reported a propensity score matching study of drainless minimally invasive lobectomy for lung cancer, which showed less postoperative pain without major complications [15]. However, the quality of surgery in the long term remains uncertain. This study aimed to investigate the perioperative outcomes, 3-year overall survival (OS), and disease-free survival (DFS) rates of patients who underwent the drainless minimally invasive anatomical lung resection surgery for pulmonary malignancies.

## Methods

### Ethical statement

This study was approved by the Research Ethics Committee of National Taiwan University Hospital, Taipei, Taiwan (202308094RINC) and was conducted in accordance with the “Ethical Principles for Medical Research Involving Human Subjects” outlined in the Declaration of Helsinki and amended by the World Medical Association. The requirement for informed consent was waived owing to the retrospective nature of this study.

### Study population

**Fig. 1:**
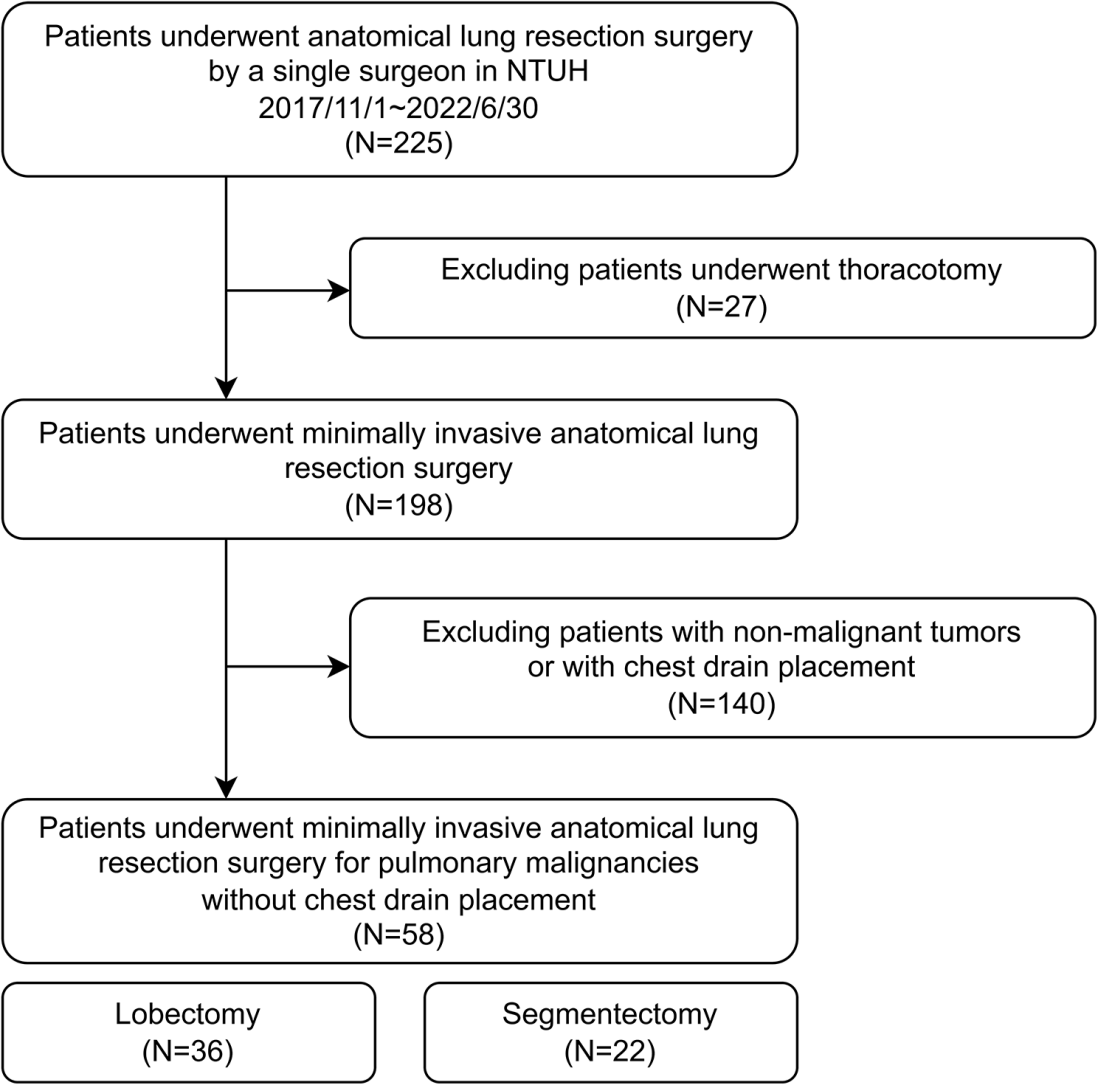
Flow chart of patient enrollment. NTUH: National Taiwan University Hospital

The enrollment criteria are shown in Fig. 1. This retrospective cohort study included 58 patients who underwent drainless minimally invasive lobectomy and segmentectomy (36 underwent lobectomy; 22 underwent segmentectomy) for pulmonary malignancies by a single surgeon in National Taiwan University Hospital between November 1, 2017, and June 30, 2022. The five-factor modified frailty index (mFi-5) was introduced to demonstrate the patients’ frailty that may influence outcomes after lung resection surgery [16]. The indications for pulmonary lobectomy were clinical T1–T3, N0–N1, or resectable N2 disease and the absence of distant metastasis, as suggested by the guidelines [17]. Segmentectomy was performed in selected patients with clinical T1a-T1b disease without nodal and distant metastases for preserving pulmonary function [18]. The practice of omitting chest drain placement was initiated in November 2017 using the following criteria: (1) patients without history of ipsilateral thoracic surgery; (2) those without inflammatory disease requiring prolonged antibiotic treatment or hospitalization in the ipsilateral lung; (3) those without history of severe pulmonary emphysema; and (4) those with sufficient pulmonary reserve [15]. 

### Outcome measurement

The study’s primary endpoint was all-cause mortality. Patients were monitored until they achieved the primary endpoint or until 16 October 2024. The primary outcome was the 3-year OS rate. Secondary outcomes were 3-year DFS rate, lymph node dissection stations and number, resection margin, postoperative pulmonary complications, pain score on POD 0, 1, and 2, and postoperative length of stay.

### Surgery and drainless technique

Minimally invasive thoracic surgery was performed using video-assisted thoracoscopic surgery (VATS) and robotic-assisted thoracoscopic surgery (RATS). VATS was performed using a 3 cm single incision in the 5th or 6th intercostal space at the anterior axillary line. RATS was performed using the Da Vinci Si or Xi robotic-assisted system with three robotic arms and a 3 cm utility incision along the anterior costophrenic region as the assistant port and for specimen retraction. The wound was extended according to the specimen size during retraction. Systemic or selective lymph node dissection was performed in all patients. 4 − 0 polypropylene (PROLENE^®^; Ethicon, US) was used to close the visible raw surface of the remaining lobe if it was created during fissure dissection. After ensuring no air leakage in the water-sealing test, a Jackson-Pratt drain (CV-1107 silicone CWV drain; Besmed Health Business Corporation, Taiwan) was placed via the working port, and the lung was reventilated under direct vision of the thoracoscope. Patients with air leaks during the water-sealing test were treated with the insertion of a small-diameter chest drain and excluded from the drainless cohort. The wound was then closed using 2 − 0 polyglactin (Vicryl^®^, Ethicon, US) continuously. During wound closure, the drain was connected to the Jackson–Pratt ball (CW-1S150 CWV reservoir 150 mL; Besmed Health Business Corporation, Taiwan) to maintain negative intrapleural pressure. The Jackson–Pratt drain was then stably retracted slowly for 2 min and removed if there was no air leakage. The Jackson–Pratt drain was maintained as a chest drain for patients with persistent air leaks during drain retraction [9, 11, 15]. 

### Postoperative management and follow-up

Within the postoperative period, chest radiography was performed 5 h after surgery, and another was performed on postoperative day (POD) 1 to evaluate air leaks and the accumulation of pleural effusion. Oral celecoxib and acetaminophen were administered once patients resumed oral intake. Intravenous morphine, tramadol, nalbuphine, or ketorolac were prescribed to patients on demand for breakthrough pain [15]. Grade III pulmonary complication was defined as any complication requiring chest drainage procedure or reoperation within two weeks according to the Clavien-Dindo classification. Adjuvant systemic therapy was administered to patients with cancer staged higher than IB, in accordance with established guidelines. [17] All patients underwent computed tomography and tumor marker assessments biannually for the first five years, followed by annual evaluations thereafter. ^18^Fluorodeoxyglucose positron emission tomography/computed tomography was utilized to assess disease extent in cases of suspected recurrence. Histological confirmation of recurrence was obtained via surgical, percutaneous, or endoscopic biopsy.

### Data collection and statistical analysis

Patients’ characteristics (age, sex, smoking history, mFi-5, forced expiratory volume in 1 s, forced vital capacity, lung cancer stage, and pathology), perioperative outcomes (operative procedure, lymph node dissection stations and number, and resection margin), postoperative outcomes (pain, complications, grade III pulmonary complications and management, length of stay), long-term outcomes (recurrence, death) were retrieved from the chart review. Statistical analyses were performed using SPSS Statistics (version 27.0; IBM, Armonk, NY, US). Continuous data are presented as median (interquartile range), and categorical data are presented as number (%). The 3-year OS and DFS rates were assessed using the Kaplan–Meier method.

## Results

**Fig. 2:**
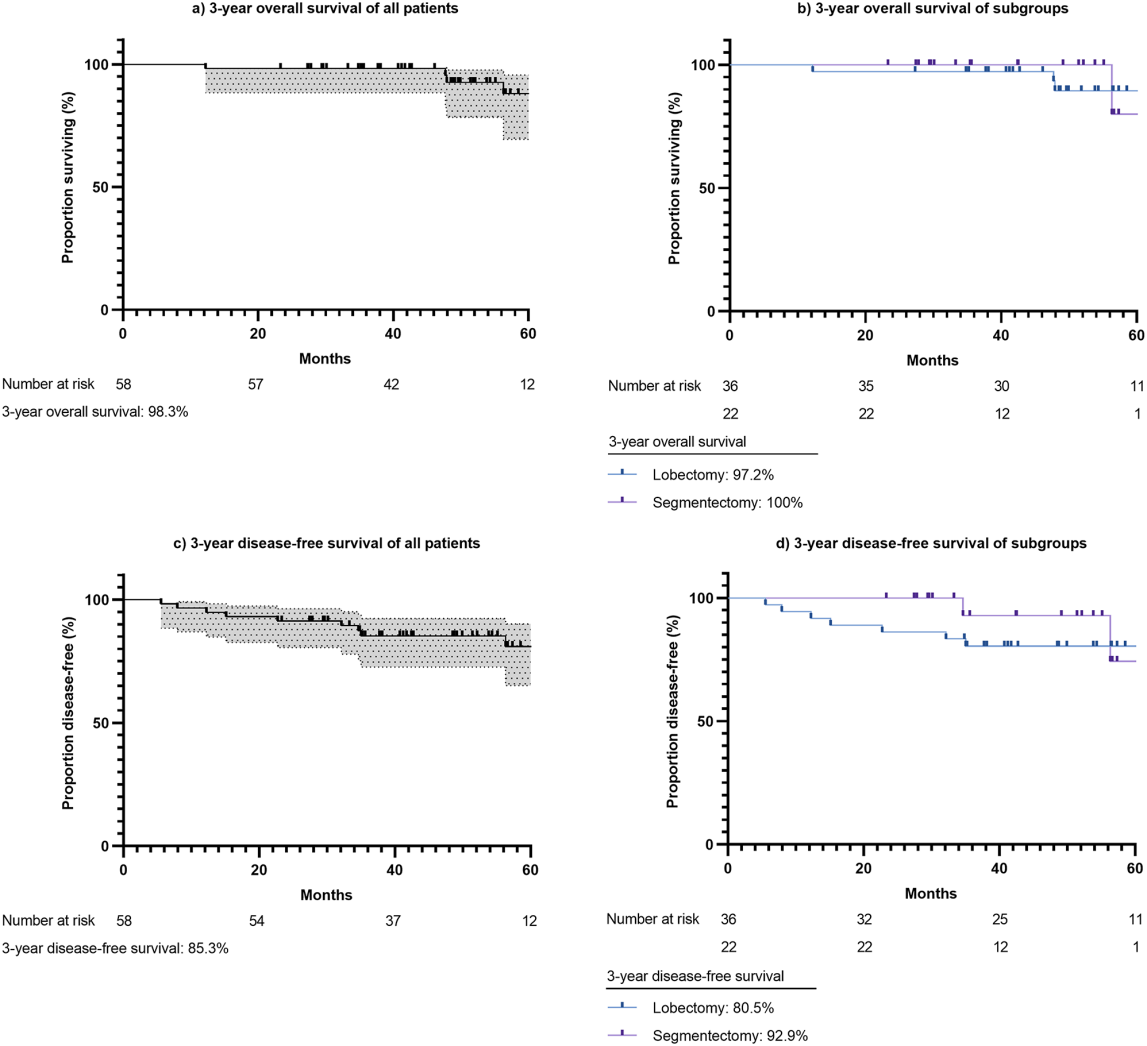
(**a**) 3-year overall survival of all patients who underwent drainless minimally invasive anatomical lung resection surgery, (**b**) 3-year overall survival of drainless minimally invasive lobectomy and segmentectomy subgroups, (**c**) 3-year disease-free survival of all patients who underwent drainless minimally invasive anatomical lung resection surgery, and (**d**) 3-year disease-free survival of drainless minimally invasive lobectomy and segmentectomy subgroups

The clinical characteristics of the 58 patients are presented in Table 1. The median age was 64 (56–69) years. Most patients were females (76%) and non-smokers (81%). The median mFi-5 was 1 (0–1). Most patients had primary lung cancer (98%), four of whom had stage 0 (7%), 43 had stage I (74%), seven had stage II (12%), and three had stage III (5%). One of the 58 patients (2%) had colorectal cancer metastasis and underwent the right segment 6 segmentectomy. Perioperative and early postoperative outcomes are presented in Table 2. The median lymph node dissection stations was 4 (4–5) and the number was 17 (8–25). The resection margins were mostly free (98%). Grade III pulmonary complications were noted in five (9%) patients, of whom one (2%) underwent pigtail catheter insertion due to pneumothorax and four (7%) underwent single thoracentesis due to residual air and pleural effusion. No patient underwent an emergent chest drainage procedure due to tension pneumothorax. The median maximal pain scores were 2 (1–2) on POD 0, 2 (1–2) on POD 1, and 1 (1–2) on POD 2. The postoperative length of stay was 4 (3–5) days. The Kaplan–Meier curves for OS and DFS rates are shown in Fig. 2. The 3-year OS rate was 98.3% in all patients with a median follow-up duration of 51.8 months and 97.2% and 100% in the lobectomy and segmentectomy subgroup, respectively. The 3-year DFS rates were 85.3% in all patients and 80.5% and 92.9% in the lobectomy and segmentectomy subgroups, respectively.

**Table 1:** Patients’ characteristics

Variables	*N* = 58
Age (yr)	64 (56–69)
Sex	
Male	14 (24%)
Female	44 (76%)
Smoking history	
Ever-smoker	11 (19%)
Non-smoker	47 (81%)
mFi-5^1^	1 (0–1)
Pulmonary function test	
FEV1 (% of predicted)	111 (100–125)
FVC (% of predicted)	109 (95–121)
Lung cancer stage	
0	4 (7%)
I	43 (74%)
II	7 (12%)
III	3 (5%)
Others^2^	1 (2%)
Pathology	
Adenocarcinoma	50 (86%)
Squamous cell carcinoma	3 (5%)
Other lung malignancies^3^	4 (7%)
Metastasis^2^	1 (2%)

Continuous data are presented as median (interquartile range) and categorical data are presented as number (%). FEV1, forced expiratory volume in 1 s; FVC, forced vital capacity; mFi-5, five-factor modified frailty index

^1^A surrogate of frailty that may affect outcomes after lung resection surgery

^2^One patient with colorectal adenocarcinoma metastasis

^3^One patient with lymphoepithelioma-like carcinoma and three patients with typical carcinoid tumor

**Table 2:** Perioperative and early postoperative outcomes

Variables	*N* = 58
Operative method	
Segmentectomy	22 (38%)
Lobectomy	36 (62%)
Lymph node dissection stations	4 (4–5)
Lymph node retrieval number	17 (8–25)
Resection margin	
Uninvolved	57 (98%)
Involved	1 (2%)
Grade III pulmonary complication^1^	5 (9%)
Pneumothorax	4 (7%)
Pleural effusion	1 (2%)
Other complication	2 (4%)
Pneumonia	1 (2%)
Empyema	0 (0%)
Arrythmia	1 (2%)
Management of grade III pulmonary complication	5 (9%)
Single thoracentesis	4 (7%)
Pigtail catheter insertion	1 (2%)
Postoperative pain	
Day 0	2 (1–2)
Day 1	2 (1–2)
Day 2	1 (1–2)
Length of stay (d)	4 (3–5)

Continuous data are presented as median (interquartile range) and categorical data are presented as number (%)

^1^Grade III pulmonary complication was defined as any complication requiring chest drainage procedure or reoperation within two weeks according to the Clavien-Dindo classification

### Discussion

In this study, we demonstrated satisfactory long-term OS and DFS rates after drainless minimally invasive anatomical lung resection surgery for pulmonary malignancies, in addition to early postoperative outcomes. The quality of surgery was assessed based on perioperative outcomes, including the lymph node dissection stations and number, resection margin, as well as long-term survival rates, including the OS and DFS rates.

The placement of chest drains after thoracic surgery has been a routine practice since the 1950s. As surgical techniques have evolved, the trend has shifted from double chest drains to a single chest drain after lobectomy [19]. While the thoracic ERAS guidelines recommend a single small-diameter chest drain with early removal, the latest studies showed the feasibility and safety of omitting chest drains from selected patients after wedge resection [1–11]. Current literature regarding the omission of chest drains after anatomical lung resection remains limited. In 2013, Ueda et al. reported the first cohort in which chest tube drainage was omitted after thoracoscopic major lung resection. In their series, chest tube drainage was omitted in 29 (58%) eligible patients and there were no adverse events during hospitalization. Furthermore, the omission of chest tube drainage was associated with reduced pain and postoperative length of stay [12, 13]. The same group published a retrospective study on the preservation of early postoperative physical function after drainless thoracoscopic major lung resection in 2019 [14]. Our group has initiated the drainless technique for minimally invasive anatomical lung resection since November 2017 and has reported a 1:1 propensity score matching study of 26 paired patients undergoing lobectomy. This result is consistent with that of Ueda et al., suggesting better pain control in the early postoperative period with no major complications [15]. Despite no randomized controlled comparative study of the drainless technique and common practice, its safety and feasibility, as well as other additional early postoperative benefits for drainless anatomical lung resection, have been demonstrated in previous retrospective studies [12–15]. However, the final examination of the quality of surgery relies on long-term outcomes that have never been explored. Despite controversies regarding the extent of lymphadenectomy in lung cancer surgery, the latest evidence still supports lymph node mapping during sublobar resection for early lung cancer [18, 20–22]. According to the current guidelines, anatomical pulmonary resection is preferred for the majority of patient with lung cancer. Sublobar resection requires a parenchymal resection margin ≥ 2 cm or the size of the nodule. A minimum of three N2 stations sampled or complete lymph node dissection was suggested [17, 21, 22]. However, a certain amount of pleural effusion is often produced after lymph node dissection and sampling, and it increases with the extent of the procedure [23]. This may hinder surgeons from performing adequate lymph node dissection or sampling for drainless procedure. None of the previous studies reported the results of lymph node dissection stations and number. The present study demonstrates the possibility that drainless minimally invasive anatomical resection surgery for pulmonary malignancies with adequate lymph node dissection can offer satisfactory long-term outcomes in addition to its early postoperative benefit for patients once the surgical principles for lung cancers are followed. To the best of our knowledge, this study is the first to demonstrate the quality of surgery based on perioperative and long-term outcomes of drainless minimally invasive anatomical lung resection surgery for pulmonary malignancies.

Our study has several limitations. First, this was a single-arm, retrospective cohort study, and a comparison between the drainless technique and common practice was not possible. Second, the cohort was highly selected and may not be applicable to the general population undergoing minimally invasive anatomical lung resection surgery. Finally, patients with previous ipsilateral thoracic surgery, moderate to severe inflammatory lung disease, severe pulmonary emphysema, and insufficient pulmonary reserve were excluded. This might lead to more favorable outcomes; therefore, the results should be interpreted carefully [24, 25]. 

In conclusion, the drainless technique is safe and feasible for minimally invasive anatomical lung resection surgery for pulmonary malignancies in terms of early postoperative and long-term outcomes. However, further randomized controlled comparative studies of the drainless technique and common practices are still required.

## Data Availability

The authors confirm that the data generated and analyzed during this study and the raw data are available from the corresponding author, upon reasonable request.
